# Obesity Phenotypes and Electrocardiographic Characteristics in Physically Active Males: CHIEF Study

**DOI:** 10.3389/fcvm.2021.738575

**Published:** 2021-10-13

**Authors:** Yu-Kai Lin, Kun-Zhe Tsai, Chih-Lu Han, Yen-Po Lin, Jiunn-Tay Lee, Gen-Min Lin

**Affiliations:** ^1^Department of Internal Medicine, Hualien Armed Forces General Hospital, Hualien City, Taiwan; ^2^National Defense Medical Center, Institute of Medical Sciences, Taipei, Taiwan; ^3^Department of Neurology, National Defense Medical Center, Tri-Service General Hospital, Taipei, Taiwan; ^4^Department of Dentistry, National Defense Medical Center, Tri-Service General Hospital, Taipei, Taiwan; ^5^Department of Medicine, Taipei Veterans General Hospital, Taipei, Taiwan; ^6^Department of Critical Care Medicine, Taipei Tzu Chi Hospital, New Taipei City, Taiwan; ^7^Department of Internal Medicine, National Defense Medical Center, Tri-Service General Hospital, Taipei, Taiwan

**Keywords:** aerobic fitness, electrocardiography, metabolic syndrome, obesity, military males, left ventricular hypertrophy

## Abstract

**Background:** Metabolically unhealthy obesity (MUO) has been associated with surface electrocardiographic (ECG) left ventricular hypertrophy (LVH), left atrial enlargement (LAE), and inferior *T* wave inversions (TWI) in the middle- and old-aged populations. However, the relationship between obesity phenotypes and these ECG abnormalities in physically active young adults is yet to be determined.

**Methods:** A total of 2,156 physically active military males aged 18–50 in Taiwan were analyzed. Obesity and metabolically unhealthy status were, respectively, defined as the body mass index ≥27 kg/m^2^ and the presence of metabolic syndrome based on the ATPIII criteria for Asian male adults. Four groups were classified as the metabolically healthy non-obesity (MHNO, *n* = 1,484), metabolically unhealthy non-obesity (MUNO, *n* = 86), metabolically healthy obesity (MHO, *n* = 376), and MUO (*n* = 210). ECG-LVH was based on the Sokolow–Lyon and Cornell voltage criteria, ECG-LAE was defined as a notched *P* wave ≥0.12 s in lead II or a notch of ≥0.04 s, and inferior TWI was defined as one negative *T* wave axis in limb leads II, III, or aVF. Physical performance was evaluated by time for a 3-km run. Multiple logistic regression analysis with adjustment for age, smoking, alcohol drinking, and physical performance was utilized to investigate the associations between obesity phenotypes and the ECG abnormalities.

**Results:** As compared to MHNO, MUNO, MHO, and MUO were associated with lower risk of Sokolow–Lyon-based ECG-LVH [odds ratios (OR) and 95% confidence intervals: 0.80 (0.51–1.25), 0.46 (0.36–0.58), and 0.39 (0.28–0.53), respectively; *p* for trend <0.001], and with greater risk of ECG-LAE [OR: 0.87 (0.44–1.72), 2.34 (1.77–3.10), and 3.02 (2.13–4.28), respectively; *p* for trend <0.001] and inferior TWI [OR: 2.21 (0.74–6.58), 3.49 (1.97–6.19), and 4.52 (2.38–8.60), respectively; *p* for trend <0.001]. However, no associations between obesity phenotypes and Cornell-based ECG-LVH were found.

**Conclusion:** In physically active young males, obesity was associated with higher risk of ECG-LAE and inferior TWI, whereas the risk between obesity and ECG-LVH might vary by the ECG criteria, possibly due to a high prevalence of exercise induced-LVH in military and greater chest wall thickness in obesity. The cardiovascular prognosis of ECG-LVH in physically active obese adults requires further study.

## Introduction

Obesity has increased dramatically over the past few decades, which results in main public health issues because it is a major factor contributing to the global burden of chronic diseases, including type 2 diabetes mellitus, coronary heart disease, sleep-breathing disorders, and certain types of cancer (1). Obesity was defined initially according to body mass index [BMI] (2). However, BMI could not precisely estimate the visceral adiposity, ectopic fat deposition, and the risk of future obesity associated atherosclerosis and cardiometabolic cormobilities (1, 3). In the 1950s, Professor Jean Vague showed that the obese with different body fat distribution may have different propensity to develop diabetes or coronary heart disease (4). Recently, the concepts of metabolically healthy obesity (MHO) and metabolically unhealthy obesity (MUO) have been established (5).

MUO is an obesity phenotype that 70–90% of obese individuals belong to this category and MHO is another phenotype that the prevalence is 10–30% in obese individuals and more common in women than in men and decreased with age (5). Most studies have defined MHO as the absence of metabolic disturbances including insulin resistance, impaired glucose metabolism, type 2 diabetes, and dyslipidemia (5, 6). On the other hand, the body fat disposition in MUO persons is located in ectopic areas including visceral tissues and the liver, which leads to abdominal obesity and increases the future risk of cardiovascular disease and type 2 diabetes (7). Previous literatures have reported that MUO individuals have higher risk of developing cardiometabolic diseases as compared to individuals with MHO (8, 9).

Obesity has been reported with an association with some electrocardiographic (ECG) abnormalities such as inferior *T* wave inversions (TWI) (10), left ventricular hypertrophy (LVH) (11, 12), and left atrial enlargement (LAE) (12) in the middle- and old-aged general populations. For a few ECG markers like prolonged QT interval (11), Ahmad et al. revealed an association with metabolic syndrome rather than obesity. The US third national health and nutrition examination survey (13) also demonstrated the prevalence of ECG-LVH highest in MUO and lowest in metabolically healthy non-obese individuals (MHNO) (11), which was in line with the reports for echocardiographic LVH in obesity (14, 15). However, the association between obesity and echocardiographic LVH decreased much and was reversed with adjustment for the baseline blood pressure (BP) levels (15). It was clear that metabolically healthy and unhealthy obesity phenotypes (lean and obesity) and other confounding factors, such as different lifestyles, substance use habits, and physical fitness affecting the ECG abnormalities, should be taken into account. Therefore, we conducted a study in physically active young males to examine the relationships between obesity phenotypes and the ECG abnormalities.

## Methods

### Study Population

The data in this study was obtained from the Cardiorespiratory Fitness and Hospitalization Events in Armed Forces (CHIEF) study in Eastern Taiwan (16). The protocol and research design of the CHIEF study have been described in detail previously (17–22). In brief, this study included 2,567 military individuals, aged 18–50 years, who underwent the annual health examinations including a self-report questionnaire survey, which included demographic factors, medical history, cigarette smoking habits (current vs. former/never), and alcohol intake status (current vs. former/never) in the past 6 months in the Hualien Armed Forces General Hospital, and performed the 3-km run test at the Military Physical Training and Testing Center in Hualien in 2014. As there were only 8 female MHO samples defined as BMI ≥ 27 kg/m^2^ and waist circumference <80 cm in metabolic syndrome, all female cases (*n* = 411) were excluded for a small sample size which had insufficient power to be analyzed, and the male subjects (*n* = 2,156) were left for the analyses. The study protocol was approved by the Institutional Review Board of the Mennonite Christian Hospital (No. 16-05-008) in Taiwan and the written informed consents were obtained from all subjects.

### Definitions of Obesity Phenotypes and Metabolic Syndrome

For Asian male adults, obesity was defined as BMI ≥ 27 kg/m^2^ according to the Taiwan's Health Promotion Administration guideline (23). Metabolic syndrome was diagnosed as having the following three or more features: (1) abdominal obesity: waist circumference ≥90 cm in men (ethnic-specifically for Chinese); (2) elevated fasting serum triglycerides ≥150 mg/dL or on lipid-lowering therapy; (3) low high-density lipoprotein cholesterol <40 mg/dL in men; (4) increased systolic BP ≥ 130 mmHg and/or diastolic BP ≥ 85 mmHg or on antihypertensive therapy; (5) high fasting plasma glucose ≥100 mg/dL or on antidiabetic therapy, according to an updated clinical criteria of International Diabetes Federation (24). Four groups were thus classified into MHNO [BMI <27.0 kg/m^2^ and metabolic syndrome (–), *n* = 1,484], MUNO [BMI <27.0 kg/m^2^ and metabolic syndrome (+), *n* = 86], MHO [BMI ≥ 27.0 kg/m^2^ and metabolic syndrome (–), *n* = 376], and MNO [BMI ≥ 27.0 kg/m^2^ and metabolic syndrome (+), *n* = 210].

### Measurements

Body height, body weight, waist circumference, and BP were measured in the standard manner. Physical examinations were performed by experienced nurses and physicians. Body height was measured in meters (without shoes), and body weight was measured in kilograms (take off heavy clothes). The definition of BMI was calculated as body weight in kilograms divided by the square of body height in meters. Waist circumference was measured at the midpoint between the rib cage and the iliac crest in the standing position. Hemodynamic status of BP was automatically measured once over the right upper arm in a sitting posture, after a rest for at least 15 min by the FT201 blood pressure monitor (Parama-Tech Co., Ltd, Fukuoka, Japan), which used the oscillometric method *via* the cuff inflated to exceed systolic BP and then deflated at a rate of 3 mmHg per second. The procedure of BP measurement was operated by an experienced outpatient nurse. If the BP level was too high to be detected, the whole procedure would be repeated for three times with a 15-min interval, and the latest two measurements were averaged to be the final level. Overnight fasting blood specimens were collected from each individual to measure serum concentrations of total cholesterol, high-density lipoprotein, low-density lipoprotein, triglycerides, and fasting glucose on an auto analyzer (AU640, Olympus, Kobe, Japan).

### Surface Electrocardiography

Resting 12-lead ECGs were obtained by the Schiller AG CARDIOVIT MS-2015 (Baar, Switzerland). If the quality of ECG report was not visually interpretable, the technician would repeat for a new one. The ECG analysis was achieved by an automated process and interpreted by a board-certified cardiologist. ECG-LVH was diagnosed if (S-V1 or S-V2 + R-V5 or R-V6) ≥35 mm, according to the Sokolow–Lyon voltage criteria (25) and if (R-aVL + S-V3) ≥18 mm, on the basis of the Cornell voltage criteria specially for Asian young male adults (26). ECG-LAE was diagnosed as a notched *P* wave ≥0.12 s in lead II or a notch of ≥0.04 s (27). Inferior TWI was defined as at least one negative *T* wave axis in limb leads II, III, or aVF. ECG-RVH was defined if (R-V1 + S-V5 or S-V6) >10.5 mm according to the Sokolow–Lyon voltage criteria (28) or if (1) R/S ratio of V1 >1 or R/S ratio of V5 or V6 <1, or (2) R-V1 >6 mm, based on the Myers et al. criteria (29).

### Statistical Analysis

Clinical characteristics of the four obesity phenotypes (MHNO, MUNO, MHO, and MUO) were presented as mean ± standard deviation (SD) for continuous variables and compared by analysis of variance (ANOVA) among four groups. Numbers and percentage (%) reported for categorical variables were compared by chi-squared test or Fisher's exact-test among the four groups. Multiple logistic regression analysis model was utilized to compute odds ratio (OR) and 95% confidence interval (CI) for the association of the four obesity phenotypes MHNO (reference), MUNO, MHO, and MUO with ECG-LVH, ECG-LAE, and inferior TWI, respectively. Model was adjusted for age, smoking status, alcohol intake status, and the cardiorespiratory fitness level assessed by time for a 3,000-m run. In addition, multiple logistic regression analysis was utilized to examine the associations of the ECG characteristics that were significantly different across four obesity phenotypes and metabolic syndrome components with ECG-LVH in those without and with obesity. Finally, multiple logistic regression analysis was used to identify the independent factors (age, obesity, metabolic syndrome, systolic BP, diastolic BP, heart rate, smoking status, alcohol consumption status, and physical fitness) of ECG-LVH, ECG-LAE, and inferior TWI, respectively. A 2-tailed value of *p* <0.05 was considered significant. All statistical analyses were performed using with SPSS statistical software (IBM Corp. Released 2013. IBM SPSS statistics for windows, version 22.0. Armonk, NY: IBM Corp.).

## Results

### Baseline Group Characteristics

Table 1 shows the characteristics of each group stratified by obesity phenotypes. The prevalence of MHNO, MUNO, MHO, and MUO was 68.8, 4.0, 17.4, and 9.7%, respectively. All variables were statistically significant across obesity phenotypes except alcohol intake habits. Participants with obesity (MHO and MUO) were older and had lower levels of cardiorespiratory fitness.

**Table 1:** Clinical characteristics of four obesity phenotypes classified by body mass index and metabolic syndrome.

	**Non-obesity**		**Obesity**		
**ECG characteristics**	**Metabolically healthy**	**Metabolically unhealthy**	**Metabolically healthy**	**Metabolically unhealthy**	***p*** **-value**
	**(*N* = 1,484)**	**(*N* = 86)**	**(*N* = 376)**	**(*N* = 210)**	
Age (years)	26.77 ± 5.63	30.56 ± 5.68	28.55 ± 5.78	31.38 ± 5.76	<0.001
Body mass index (kg/m^2^)	23.02 ± 2.38	25.13 ± 1.64	29.20 ± 1.86	30.26 ± 2.61	<0.001
(Range: min–max)	(15.87–26.99)	(19.47–26.94)	(27.00–37.03)	(27.01–43.28)	
Waist circumference (cm)	79.14 ± 7.27	87.54 ± 5.48	92.97 ± 6.47	97.42 ± 6.67	<0.001
Prior history of hypertension	59 (4.0)	11 (12.8)	38 (10.1)	59 (28.1)	<0.001
Prior history of hyperlipidemia	199 (13.4)	27 (31.4)	94 (25.0)	69 (32.9)	<0.001
Systolic blood pressure (mmHg)	116.44 ± 11.58	128.81 ± 13.84	123.32 ± 13.43	131.53 ± 14.26	<0.001
Diastolic blood pressure (mmHg)	69.07 ± 9.26	77.13 ± 10.99	72.24 ± 10.66	78.85 ± 12.28	<0.001
**Blood test**					
Total cholesterol (mg/dL)	166.39 ± 31.22	185.43 ± 43.03	179.11 ± 30.39	191.30 ± 40.38	<0.001
HDL-C (mg/dL)	50.48 ± 9.77	38.49 ± 7.94	48.21 ± 8.14	39.92 ± 7.56	<0.001
LDL-C (mg/dL)	100.75 ± 27.73	112.19 ± 32.29	114.70 ± 27.16	120.91 ± 31.55	<0.001
Triglycerides (mg/dL)	87.85 ± 49.83	246.06 ± 167.17	104.30 ± 60.01	209.08 ± 144.10	<0.001
FPG (mg/dL)	92.48 ± 9.50	103.37 ± 15.92	92.91 ± 7.32	102.40 ± 24.31	<0.001
Blood pressure ≥130/85 mmHg	243 (15.7)	52 (60.5)	125 (33.2)	137 (65.2)	<0.001
Serum triglycerides ≥150 mg/dL	136 (8.8)	69 (80.2)	46 (12.2)	142 (67.6)	<0.001
HDL-C <40 mg/dL	139 (9.0)	61 (70.9)	38 (10.1)	119 (56.7)	<0.001
Waist circumference ≥90 cm	87 (5.6)	36 (41.9)	266 (70.7)	202 (96.2)	<0.001
FPG ≥100 mg/dL	218 (14.1)	56 (65.1)	45 (12.0)	112 (53.3)	<0.001
**Unhealthy behavior**					
Current cigarette smoker	634 (42.7)	49 (57.0)	165 (43.9)	100 (47.6)	0.04
Current alcohol drinker	704 (47.4)	43 (50.0)	189 (50.3)	106 (50.5)	0.67
3,000-m running (s)	849.98 ± 76.74	907.15 ± 101.10	908.61 ± 105.32	946.96 ± 106.35	<0.001
Top 25% performance	664 (44.7)	30 (34.9)	99 (26.3)	65 (31.0)	<0.001
Middle 50% performance	589 (39.7)	29 (33.7)	146 (38.8)	50 (23.8)	
Bottom 25% performance	231 (15.6)	27 (31.4)	131 (34.8)	95 (45.2)	

*Continuous variables are expressed as mean ± standard deviation, and categorical variables as n (%)*.

*ECG, electrocardiography; HDL-C, high-density lipoprotein cholesterol; LDL-C, low-density lipoprotein cholesterol; LVH, left ventricular hypertrophy; FPG, fasting plasma glucose*.

*Obesity was defined as the body mass index ≥27.0 kg/m^2^*.

*Metabolically health was defined as absence of metabolic syndrome, according to the International Diabetes Federation criteria for Asian male adults*.

Table 2 reveals the ECG characteristics of each group by obesity phenotypes. Across the four groups, MHNO had the lowest heart rate, PR interval, and QTc interval. In addition, MHNO had the greatest QRS axis and was more likely to have Sokolow–Lyon-based ECG-LVH, sinus bradycardia, right axis deviation, and prolonged QT interval. However, there were no significant differences in the prevalence of Cornell-based ECG-LVH and ECG-RVH. On the contrary, the obese groups were more likely to have ECG-LAE, first degree atrioventricular block, and inferior TWI.

**Table 2:** Electrocardiographic characteristics of four obesity phenotypes classified by body mass index and metabolic syndrome.

	**Non-obesity**		**Obesity**		
**ECG characteristics**	**Metabolically healthy**	**Metabolically unhealthy**	**Metabolically healthy**	**Metabolically unhealthy**	***p*** **-value**
	**(*N* = 1,484)**	**(*N* = 86)**	**(*N* = 376)**	**(*N* = 210)**	
Heart rate (beats/min)	65.68 ± 10.40	68.08 ± 11.53	66.44 ± 10.34	72.07 ± 12.81	<0.001
P duration (ms)	105.98 ± 14.19	109.43 ± 13.48	106.45 ± 15.65	107.97 ± 15.66	0.06
PR interval (ms)	156.29 ± 19.65	157.18 ± 14.52	159.78 ± 20.82	161.51 ± 21.44	<0.001
QRS duration (ms)	97.81 ± 10.78	97.56 ± 12.10	97.66 ± 10.04	97.42 ± 11.91	0.96
QTc interval (ms)	388.49 ± 21.84	392.01 ± 22.44	393.77 ± 26.14	401.68 ± 25.17	<0.001
QRS axis (degree)	67.89 ± 29.11	50.85 ± 36.75	51.20 ± 33.26	44.36 ± 33.71	<0.001
Sokolow-Lyon based ECG-LVH (%)	890 (60.0)	42 (48.8)	145 (38.6)	66 (31.4)	<0.001
Cornell based ECG-LVH (%)	213 (14.4)	11 (12.8)	59 (15.7)	38 (18.1)	0.47
ECG-RVH (%)	226 (15.2)	6 (7.0)	61 (16.2)	27 (12.9)	0.13
Sinus bradycardia (%)	432 (29.1)	19 (22.1)	88 (23.4)	32 (15.2)	<0.001
Ectopic P rhythm (%)	58 (3.9)	4 (4.7)	14 (3.7)	11 (5.2)	0.79
ECG-LAE (%)	205 (13.8)	10 (11.6)	99 (26.3)	64 (30.5)	<0.001
First degree atrioventricular block (%)	37 (2.5)	0 (0.0)	16 (4.3)	15 (7.1)	0.001
Left axis deviation (%)	19 (1.3)	3 (3.5)	7 (1.9)	6 (2.9)	0.16
Right axis deviation (%)	186 (12.5)	8 (9.3)	24 (6.4)	13 (6.2)	0.001
Complete right bundle branch block (%)	51 (3.4)	4 (4.7)	12 (3.2)	10 (4.8)	0.70
Incomplete right bundle branch block (%)	101 (6.8)	8 (9.3)	29 (7.7)	8 (3.8)	0.22
QT interval prolongation (%)	16 (1.1)	0 (0.0)	9 (2.4)	6 (2.9)	0.04
QTc interval prolongation (%)	6 (0.4)	0 (0.0)	4 (1.1)	2 (1.0)	0.32
Inferior TWI (%)	27 (1.8)	4 (4.7)	25 (6.6)	20 (9.5)	<0.001

*Continuous variables are expressed as mean ± standard deviation, and categorical variables as n (%)*.

*Obesity was defined as body mass index ≥27.0 kg/m^2^*.

*Metabolically health was defined as absence of metabolic syndrome, according to the International Diabetes Federation criteria for Asian male adults*.

*Sokolow-Lyon ECG based-LVH was diagnosed if (S-V1 or S-V2 + R-V5 or R-V6) ≥35 mm and Cornell ECG-based LVH was defined if R-aVL + S-V3 ≥18 mm, specifically for Asian young male adults*.

*ECG, electrocardiography; LAE, left atrial enlargement; LVH, left ventricular hypertrophy; RVH, right ventricular hypertrophy; TWI, T wave inversion*.

Table 3 reveals the results of multiple logistic regression analyses for MUNO, MHO, and MUO compared to MHNO with ECG-LVH, ECG-LAE, and inferior TWI. As compared to MHNO, MUNO, MHO, and MUO were associated with lower risk of Sokolow–Lyon-based ECG-LVH [OR and 95% CI: 0.80 (0.51–1.25), 0.46 (0.36–0.58), and 0.39 (0.28–0.53), respectively; *p* for trend <0.001] and with greater risk of ECG-LAE [OR: 0.87 (0.44–1.72), 2.34 (1.77–3.10), and 3.02 (2.13–4.28), respectively; *p* for trend <0.001] and inferior TWI [OR: 2.21 (0.74–6.58), 3.49 (1.97–6.19), and 4.52 (2.38–8.60), respectively; *p* for trend <0.001]. However, no associations between obesity phenotypes and Cornell-based ECG-LVH were found.

**Table 3:** Associations of obesity phenotypes with ECG-LVH, ECG-LAE and inferior TWI.

	**Sokolow-Lyon based ECG-LVH**		**Cornell based ECG-LVH**		**ECG-LAE**		**Inferior TWI**	
**Obesity phenotypes**	**OR (95% CI)**	***p*** **-value**	**OR (95% CI)**	***p*** **-value**	**OR (95% CI)**	***p*** **-value**	**OR (95% CI)**	***p*** **-value**
MHNO	1.00		1.00		1.00		1.00	
MUNO	0.80 (0.51–1.25)	0.32	0.79 (0.41–1.53)	0.48	0.87 (0.44–1.72)	0.68	2.21 (0.74–6.58)	0.15
MHO	0.46 (0.36–0.58)	<0.001	1.08 (0.79–1.50)	0.62	2.34 (1.77–3.10)	<0.001	3.49 (1.97–6.19)	<0.001
MUO	0.39 (0.28–0.53)	<0.001	1.19 (0.80–1.77)	0.39	3.02 (2.13–4.28)	<0.001	4.52 (2.38–8.60)	<0.001
*p*-value for trend		<0.001		0.39		<0.001		<0.001

*Data are presented as odds ratios (OR) and 95% CI (confidence intervals) using multiple logistic regression analysis*.

*Model adjusted for age, cigarette smoking status, alcohol drinking status and 3,000-m running categorical performance*.

*Sokolow-Lyon ECG based-LVH was diagnosed if (S-V1 or S-V2 + R-V5 or R-V6) ≥35 mm and Cornell ECG-based LVH was defined if R-aVL + S-V3 ≥18 mm, specifically for Asian young male adults*.

*MHNO, metabolically healthy non-obesity, defined as body mass index <27.0 kg/m^2^ and absence of metabolic syndrome, according to the International Diabetes Federation criteria for Asian male adults; MHO, metabolically healthy obesity defined as body mass index ≥27.0 kg/m^2^ and absence of metabolic syndrome; MUNO, metabolically unhealthy non-obesity defined as body mass index <27.0 kg/m^2^ and presence of metabolic syndrome; MUO, metabolically unhealthy obesity defined as body mass index ≥27.0 kg/m^2^ and presence of metabolic syndrome; ECG, electrocardiography; LAE, left atrial enlargement; LVH, left ventricular hypertrophy; TWI, T wave inversion*.

Table 4 reveals the results of multiple logistic regression analyses for specific ECG characteristics and metabolic syndrome components with ECG-LVH in those with and without obesity. In the non-obese, Sokolow–Lyon-based ECG-LVH was associated with higher risk of sinus bradycardia [OR: 1.27 (1.01–1.60)], but with lower risk of right axis deviation [OR: 0.53 (0.39–0.72)]; while Cornell-based ECG-LVH was associated with greater risk of right axis deviation [OR: 2.72 (1.91–3.87)]. ECG-LAE, first degree atrioventricular block, and inferior TWI were not associated with Sokolow–Lyonor Cornell-based ECG-LVH in both the non-obese and the obese groups except that a marginal association of ECG-LAE with Cornell-based ECG-LVH was observed in the non-obese [OR: 1.45 (0.99–2.12), *p* = 0.053]. Of the metabolic components, low high-density lipoprotein was associated with lower risk of both Sokolow–Lyon and Cornell-based ECG-LVH in the non-obese [OR: 0.70 (0.51–0.96) and 0.58 (0.35–0.96), respectively]. High BP was associated with greater risk of both Sokolow–Lyon and Cornell-based ECG-LVH in the obese [OR: 1.45 (1.01–2.07) and 1.70 (1.07–2.69), respectively]. No other metabolic syndrome components were significantly associated with the risk of ECG-LVH.

**Table 4:** Associations of specific ECG characteristics and metabolic components with ECG-LVH in the obese and non-obese.

	**Sokolow-Lyon based ECG-LVH**				**Cornell based ECG-LVH**			
	**Non-obesity**		**Obesity**		**Non-obesity**		**Obesity**	
	**OR (95% CI)**	***p*** **-value**	**OR (95% CI)**	***p*** **-value**	**OR (95% CI)**	***p*** **-value**	**OR (95% CI)**	***p*** **-value**
**ECG characteristics**								
Sinus bradycardia	1.27 (1.01–1.60)	0.04	0.89 (0.58–1.37)	0.59	1.13 (0.83–1.55)	0.43	0.85 (0.48–1.53)	0.60
Right axis deviation	0.53 (0.39–0.72)	<0.001	1.06 (0.53–2.12)	0.87	2.72 (1.91–3.87)	<0.001	1.49 (0.65–3.44)	0.34
ECG-LAE	1.02 (0.76–1.37)	0.90	1.09 (0.75–1.06)	0.64	1.45 (0.99–2.12)	0.053	1.14 (0.70–1.85)	0.61
First degree atrioventricular block	0.96 (0.49–1.88)	0.90	1.56 (0.74–3.28)	0.24	0.73 (0.25–2.08)	0.55	1.36 (0.55–3.33)	0.50
Inferior TWI	1.06 (0.51–2.21)	0.87	0.76 (0.38–1.50)	0.42	1.78 (0.76–4.19)	0.18	1.64 (0.78–3.41)	0.19
**Metabolic components**								
Blood pressure ≥130/85 mmHg	1.22 (0.93–1.59)	0.15	1.45 (1.01–2.07)	0.04	1.15 (0.80–1.63)	0.45	1.70 (1.07–2.69)	0.02
Serum triglycerides ≥150 mg/dL	1.18 (0.85–1.64)	0.32	0.86 (0.57–1.29)	0.46	0.79 (0.50–1.25)	0.31	1.21 (0.75–1.94)	0.44
HDL-C <40 mg/dL	0.70 (0.51–0.96)	0.02	0.86 (0.57–1.30)	0.48	0.58 (0.35–0.96)	0.03	0.80 (0.48–1.34)	0.39
Waist circumference ≥90 cm	0.71 (0.48–1.05)	0.08	0.70 (0.45–1.07)	0.10	1.28 (0.77–2.13)	0.33	1.45 (0.78–2.70)	0.24
FPG ≥100 mg/dL	0.99 (0.75– 1.30)	0.92	1.25 (0.84–1.87)	0.27	0.86 (0.58– 1.27)	0.44	0.80 (0.48–1.33)	0.38

*Data are presented as odds ratios (OR) and 95% CI (confidence intervals) using multiple logistic regression*.

*Model adjusted for age plus cigarette smoking status, alcohol drinking status and 3,000-m running categorical performance*.

*Sokolow-Lyon based ECG-LVH was diagnosed if (S-V1 or S-V2 + R-V5 or R-V6) ≥35 mm and Cornell based ECG-LVH was defined if R-aVL + S-V3 ≥18 mm, specifically for Asian young male adults*.

*ECG, electrocardiography; FPG, fasting plasma glucose; HDL-C, high-density lipoprotein cholesterol; LAE, left atrial enlargement; LVH, left ventricular hypertrophy; TWI, T wave inversion*.

Table 5 shows the results of multiple logistic regression model to determine the independent predictors of the ECG abnormalities. Older age, obesity, metabolic syndrome, and current smokers were protective factors of Sokolow–Lyon-based ECG-LVH [OR: 0.95 (0.93–0.97), 0.42 (0.33–0.52), 0.73 (0.55–0.99), and 0.80 (0.65–0.99), respectively], and in contrast, systolic BP and current alcohol consumer were risk factors of Sokolow–Lyon-based ECG-LVH [OR: 1.02 (1.01–1.03) and 1.48 (1.20–1.82), respectively]. However, diastolic BP was the only risk factor of Cornell-based ECG-LVH [OR: 1.02 (1.01–1.04)]. For ECG-LAE and inferior TWI, obesity was a risk factor [OR: 2.51 (1.92–3.28) and 2.73 (1.59–4.67), respectively], whereas slower heart rate was a protective factor [OR: 0.97 (0.96–0.98) and 0.98 (0.95–0.99), respectively]. Systolic BP was also a risk factor of inferior TWI [OR: 1.03 (1.00–1.05)].

**Table 5:** Associations between cardiometabolic risk factors and ECG-LVH, ECG-LAE and Inferior TWI.

	**Sokolow-Lyon based ECG-LVH**		**Cornell based ECG-LVH**		**ECG-LAE**		**Inferior TWI**	
	**OR (95% CI)**	***p*** **-value**	**OR (95% CI)**	***p*** **-value**	**OR (95% CI)**	***p*** **-value**	**OR (95% CI)**	***p*** **-value**
Age (years)	0.95 (0.93–0.97)	<0.001	1.02 (0.99–1.04)	0.15	0.99 (0.96–1.01)	0.17	1.01 (0.97–1.06)	0.65
Obesity	0.42 (0.33–0.52)	<0.001	1.09 (0.81–1.47)	0.57	2.51 (1.92–3.28)	<0.001	2.73 (1.59–4.67)	<0.001
Metabolic syndrome	0.73 (0.55–0.99)	0.04	0.87 (0.59–1.27)	0.46	1.26 (0.90–1.78)	0.18	1.27 (0.71–2.28)	0.42
Systolic blood pressure (mmHg)	1.02 (1.01–1.03)	<0.001	0.99 (0.98–1.01)	0.86	1.00 (0.99–1.02)	0.96	1.03 (1.00–1.05)	0.03
Diastolic blood pressure (mmHg)	1.00 (0.98–1.01)	0.46	1.02 (1.01–1.04)	0.006	1.01 (0.99–1.02)	0.26	1.01 (0.98–1.04)	0.68
Heart rate (beats per min)	0.99 (0.98–1.00)	0.05	1.00 (0.99–1.01)	0.91	0.97 (0.96–0.98)	<0.001	0.98 (0.95–0.99)	0.02
Current cigarette smoking	0.80 (0.65–0.99)	0.03	1.15 (0.87–1.51)	0.32	1.19 (0.91–1.54)	0.20	1.56 (0.91–2.66)	0.10
Current alcohol beverages intake	1.48 (1.20–1.82)	<0.001	1.00 (0.76–1.32)	0.99	0.92 (0.71–1.20)	0.53	0.93 (0.55–1.58)	0.77
**3,000-m running categorical levels**								
Top 25% performance	1.03 (0.84–1.27)	0.75	1.09 (0.83–1.43)	0.54	0.93 (0.71–1.21)	0.58	1.25 (0.68–2.29)	0.46
Middle 50% performance	1.00		1.00		1.00		1.00	
Bottom 25% performance	0.94 (0.74–1.20)	0.62	0.95 (0.69–1.33)	0.77	0.91 (0.67–1.24)	0.54	1.83 (0.99–3.36)	0.06

*Data are presented as odds ratios (OR) and 95% CI (confidence intervals) using multiple logistic regression*.

*Obesity was defined as body mass index ≥27.0 kg/m^2^*.

*Metabolic syndrome was defined according to the International Diabetes Federation criteria for Asian male adults*.

*Sokolow-Lyon based ECG -LVH was diagnosed if (S-V1 or S-V2 + R-V5 or R-V6) ≥35 mm and Cornell based ECG- LVH was defined if R-aVL + S-V3 ≥18 mm, specifically for Asian young male adults*.

*ECG, electrocardiography; LAE, left atrial enlargement; LVH, left ventricular hypertrophy; TWI, T wave inversion*.

## Discussion

In this study conducted in a well-controlled military environment, we found some interesting and important points. First, the prevalenceof Sokolow–Lyon-based ECG-LVH was highest in MHNO followed by MUNO, MHO, and then MNO; however, the prevalence of Cornell-based ECG-LVH was similar across the four groups. On the contrary, the prevalence of ECG-LAE and inferior TWI in the obese were higher than that in MUNO and MHNO. Second, MHO and MUO were associated with lower risk of Sokolow–Lyon-based ECG-LVH, while were with higher risk of ECG-LAE and inferior TWI with adjustments for age, cigarette smoking status, alcohol intake status, and physical performance level. There were no associations of obesity phenotypes with Cornell-based ECG-LVH. Third, greater BP was associated with higher risk of Sokolow–Lyon and Cornell-based ECG-LVH in obese participants; however, low high-density lipoprotein was associated with lower risk of Sokolow–Lyon and Cornell-based ECG-LVH in non-obese participants. For right axis deviation, there was an association for Cornell-based ECG-LVH, which was contradictory to an inverse association for Sokolow–Lyon-based ECG-LVH in the non-obese. Finally, in physically active male adults, younger age, obesity, metabolic syndrome, and cigarette smoking were independent protective factors of Sokolow–Lyon-based ECG-LVH, while greater systolic BP and alcohol consumption were independent risk factors of Sokolow–Lyon-based ECG-LVH. Diastolic BP was the only risk factor of Cornell-based ECG-LVH. Obesity was the independent factor of ECG-LAE and inferior TWI.

Prior studies reported that the components of metabolic syndrome such as high waist circumference were associated with ECG-LVH in general population (13, 15). The present study revealed that the prevalence of Sokolow–Lyon-based ECG-LVH and high level of the endurance capacity (the top 25% performance in the 3,000-m run test) were highest in MHNO and lowest in MUO among young physically active males. Obesity and metabolic syndrome were found inversely associated with Sokolow–Lyon-based ECG-LVH; however, the association was null for Cornell-based ECG-LVH. The mechanism might be reasoned in part by the distance between the heart and the ECG precordial leads is shorter and less insulated in lean individuals, leading to the electrical signals stronger than the obese individuals. Similarly, a meta-analysis showed that the prevalence of exercise-induced LVH (athlete heart) is higher in highly trained male athletes (30). Consistent to our findings, the work performed by Scharf et al. showed that interval changes in the left ventricular structure were highly correlated with an increase of high-intensity aerobic training (31). As compared with Sokolow–Lyon-based ECG-LVH, Cornell-based ECG-LVH, which utilized a limb lead voltage in the criteria to reduce the effect of chest wall thickness on the signals received in precordial leads, has shown higher accuracy to echocardiographic LVH in young males (26).

Among the components of metabolic syndrome, hyperglycemia and elevated BP were regarded as independent risk factors for the development of LVH (32–34). Similarly in the present study, we observed an association between higher BP and ECG-LVH in the obesity subgroup. However, there was no association of higher BP with ECG-LVH in non-obese participants. These findings suggested a marked attenuation in association between hypertension and ECG-LVH as the impact of lean body mass was taken into account among physically active obese individuals, which highlights a possibility of the presence of pathological LVH in this population. Consistent with our findings, Manolio et al. demonstrated that moderate and high alcohol consumption was positively associated with left ventricular mass in men (35). It is believed that alcohol has a direct toxic effect on the myocardium through ethanol or its major metabolite, acetaldehyde (36).

With regard to the ECG-LAE and inferior TWI, the present study findings for physically active young adults were consistent to the results in prior studies for middle- and old-aged general populations that the obese ones have higher risk of ECG-LAE and inferior TWI (10–12). Eisenstein et al. have uncovered that inferior TWI was possibly due to leftward and horizontal displacement of the base of the heart in central obesity (10). In addition, both obesity and metabolic syndrome have been associated with greater *P* wave duration as compared to the thin and metabolically healthy counterparts (11, 12). However, there were some controversies for other ECG characteristics with obesity among studies. Compared to MHNO, MUO had longer QRS duration and QT interval in the US third national health and nutrition examination survey (11), but not in the epidemiology of obesity study in the Netherlands (12). In the present study, MUO had longer QTc interval but with similar length in the QRS duration. The mechanisms for the conflicting findings were not fully understood and need further investigations.

There were a number of strengths of this study. First, the study sample size had sufficient numbers of military males for analyses of the four classified groups. Second, all the military males lived in the same environment and all laboratory measurements were standardized and performed in a referral medical center to minimize the potential confounders to affect the study results. On the other hand, several limitations of this study should be considered. First, this study recruited only physically active young male individuals so that the results may not be appropriately extrapolated to the age-matched general population of males. Second, we could not avoid the presence of potential bias that affect the ECG results such as a lack of information regarding the body temperature and duration of cardiovascular risk factors presence such as hypertension, despite that the effects were small in young adults. Third, the echocardiographic data were lacking to investigate the correlation with obesity phenotypes. Finally, we employed a cross-sectional design, the causality between the metabolic healthy status, obesity, and toxic substance use, and the risk of ECH-LVH could not be clarified. For example, an association of active tobacco smoking with Sokolow–Lyon-based ECG-LVH was not fully understood, which was possibly related to a higher prevalence of current smoking in participants of thin body weight.

In conclusion, our findings suggested that in physically active young males, the obese had higher risk of ECG-LAE and inferior TWI. Although higher BP level remained the potential risk factor of ECG-LVH, both metabolic syndrome and obesity were associated with the lower risk of Sokolow–Lyon-based ECG-LVH, but not associated with Cornell-based ECG-LVH, possibly due to a high prevalence of exercise induced-physiological LVH in military and low signal strengths of greater chest wall thickness in obesity. The cardiovascular prognosis of various ECG-LVH in physically active obese adults requires further study.

## Data Availability Statement

The raw data supporting the conclusions of this article will be made available by the authors, without undue reservation.

## Ethics Statement

The studies involving human participants were reviewed and approved by the Institutional Review Board of the Mennonite Christian Hospital (No. 16-05-008) in Taiwan. The patients/participants provided their written informed consent to participate in this study.

## Author Contributions

Y-KL wrote the paper. K-ZT made the statistical analyses. Y-PL, C-LH, and J-TL raised critical comments for the paper. G-ML was the principal investigator for the study. All authors contributed to the article and approved the submitted version.

## Funding

This study was supported by the Medical Affairs Bureau Ministry of National Defense and Hualien Armed Forces General Hospital, Taiwan, under the Grants MND-MAB-110-148 and HAFGH-D-110008.

## Conflict of Interest

The authors declare that the research was conducted in the absence of any commercial or financial relationships that could be construed as a potential conflict of interest.

## Publisher's Note

All claims expressed in this article are solely those of the authors and do not necessarily represent those of their affiliated organizations, or those of the publisher, the editors and the reviewers. Any product that may be evaluated in this article, or claim that may be made by its manufacturer, is not guaranteed or endorsed by the publisher.

## Abbreviations

BP: blood pressure
CHIEF: Cardiorespiratory Fitness and Hospitalization Events in Armed Forces study
CRF: cardiorespiratory fitness
ECG: electrocardiography
MHNO: metabolically healthy non-obesity
MHO: metabolically healthy obesity
MUNO: metabolically unhealthy non-obesity
MUO: metabolically unhealthy obesity
LAE: left atrial enlargement
LVH: left ventricular hypertrophy
TWI: *T* wave inversion.
